# Uncovering Tumor‐Promoting Roles of Activin A in Pancreatic Ductal Adenocarcinoma

**DOI:** 10.1002/advs.202207010

**Published:** 2023-04-21

**Authors:** Seok‐Yeong Yu, Yi Luan, Siyuan Tang, Amirhossein Abazarikia, Rosemary Dong, Thomas C. Caffrey, Michael A. Hollingsworth, David Oupicky, So‐Youn Kim

**Affiliations:** ^1^ Olson Center for Women's Health Department of Obstetrics and Gynecology College of Medicine University of Nebraska Medical Center Omaha NE USA; ^2^ Center for Drug Delivery and Nanomedicine Department of Pharmaceutical Sciences College of Pharmacy University of Nebraska Medical Center Omaha NE USA; ^3^ Eppley Institute for Research in Cancer and Allied Diseases University of Nebraska Medical Center Omaha NE 68198 USA; ^4^ Fred & Buffett Cancer Center University of Nebraska Medical Center Omaha NE USA

**Keywords:** activin A, cancer‐associated fibroblasts, cholesterol‐modified polymeric nanoparticle, orthotopic mice, pancreatic ductal adenocarcinoma

## Abstract

Pancreatic ductal adenocarcinoma (PDAC) is one of the most lethal cancers with high incidence rates of metastasis and cachexia. High circulating activin A, a homodimer of inhibin βA subunits that are encoded by INHBA gene, predicts poor survival among PDAC patients. However, it still raises the question of whether activin A suppression renders favorable PDAC outcomes. Here, the authors demonstrate that activin A is abundantly detected in tumor and stromal cells on PDAC tissue microarray and mouse PDAC sections. In orthotopic male mice, activin A suppression, which is acquired by tumor‐targeted Inhba siRNA using cholesterol‐modified polymeric nanoparticles, retards tumor growth/metastasis and cachexia and improves survival when compared to scramble siRNA‐treated group. Histologically, activin A suppression coincides with decreased expression of proliferation marker Ki67 but increased accumulation of α‐SMA^high^ fibroblasts and cytotoxic T cells in the tumors. In vitro data demonstrate that activin A promotes KPC cell proliferation and induces the downregulation of α‐SMA and upregulation of IL‐6 in pancreatic stellate cells (PSC) in the SMAD3‐dependent mechanism. Moreover, conditioned media from activin A‐stimulated PSC promoted KPC cell growth. Collectively, our data provide a mechanistic basis for tumor‐promoting roles of activin A and support therapeutic potentials of tumor activin A suppression for PDAC.

## Graphical abstract

Activin A acts as a tumor‐promoting mediator by increasing tumor cell proliferation and by regulating *α*‐SMA expression in pancreatic stellate cells in a SMAD3‐dependent manner. Tumor activin A suppression decreases pancreatic ductal adenocarcinoma tumor growth, metastasis, and weight loss, thereby improving survival.

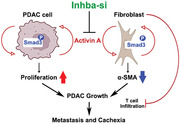

## 1 Introduction

Pancreatic ductal adenocarcinoma (PDAC) accounts for more than 90% of pancreatic cancer. *KRAS* mutations are present in nearly 95% of PDAC tumors and co‐present with *TP53* mutation in approximately 70% of PDAC tumors.^[^ ^1^ ^]^ PDAC has approximately 10% 5‐year relative survival rates and is the third leading cause of cancer‐related deaths in the US.^[^ ^2^ ^]^ The dismal prognosis pertains to few early diagnostic tools, chemoresistance, and high incidence of comorbidities including cachexia.^[^ ^3^ ^]^ Therefore, more efforts are needed towards improving PDAC prognosis by identifying prognostic factors for PDAC and their underlying mechanisms.^[^ ^4^ ^]^

Inhibin *β*A (*INHBA*) is a subunit of activins and inhibin A which are members of the transforming growth factor *β* family (TGF‐*β*). The homodimer of inhibin *β*A subunits constitutes activin A, and inhibin *β*A and inhibin *β*B (*INHBB*) form activin AB. Inhibin *β*A also heterodimerizes with inhibin *α* (*INHA*) to make inhibin A which antagonizes activin A by competing for Activin receptors.^[^ ^5^ ^]^ Activin A was first discovered from the porcine follicular fluid as an ovarian hormone.^[^ ^6^ ^]^ Activin A acts through its binding to activin type 2 receptors (ACVR2A or ACVR2B), which recruits type 1 receptors to form a receptor complex.^[^ ^7^ ^]^ Then, intracellular signaling transductions are mediated by two pathways: the canonical pathway, where SMAD3 is phosphorylated and recruits SMAD4 to acquire transcriptional activity, and the noncanonical pathway, where subfamilies of the mitogen‐activated protein kinases including p38 activate downstream molecules.^[^ ^8^ ^]^ SMAD3‐dependent signaling is indispensable for the proliferation of ovarian granulosa cells by activin A, and the downstream targets, *Inha* and *Fst*, feedback inhibits activin A‐mediated proliferation.^[^ ^9^ ^]^ The failure of antagonizing activin A has been linked to the spontaneous development of granulosa cell tumors in mice.^[^ ^10^ ^]^

Existing evidence suggests activin A as a prognostic factor for PDAC. Our group reported that serum activin A levels are significantly elevated in Stage IV PDAC patients than in healthy individuals.^[^ ^11^ ^]^ Higher activin A levels are associated with metastasis and poor survival in PDAC patients and animals.^[^ ^12^ ^]^ Correspondingly, high tumor SMAD3 expression is associated with tumor size, lymph node metastasis, and poor survival in PDAC patients.^[^ ^13^ ^]^ However, it is unclear whether activin A suppression would hold therapeutic potentials for PDAC due perhaps to a limited understanding of target cells and/or mechanisms which activin A acts through to promote PDAC. In addition, the therapeutic relevance of suppressing activin A expression in PDAC needs to be addressed.

Cellular heterogeneity is a prominent feature of PDAC and is characterized by the co‐existence of heterogenous tumor cells at different subtypes and states and interconvertible activation states of cancer‐associated fibroblasts (CAFs) which largely originate from pancreatic stellate cells (PSCs).^[^ ^14^ ^]^ CAFs are subtyped by different gene expression patterns and modulate metastasis and immune exclusion.^[^ ^14^, ^15^ ^]^ High *α*‐SMA‐expressing myofibroblastic CAFs (myCAF) form dense fibrosis, prevent metastasis, and promote T cell infiltration in genetically engineered PDAC mice.^[^ ^16^ ^]^ However, inflammatory CAFs (iCAFs), which highly express inflammatory cytokines including IL‐6, decrease T cell infiltration and tumor cell death, promoting PDAC growth.^[^ ^17^ ^]^ Therefore, PDAC progression is accounted for by not only tumor cell proliferation but also their reciprocal interaction with CAFs.

Here, we investigated the expression of inhibin *β*A protein in normal pancreas, chronic pancreatitis, premalignant pancreatic intraepithelial neoplasia (PanIN), and PDAC in humans. In orthotopic PDAC mice, we characterized the therapeutic potentials of tumor activin A on tumor growth and addressed tumor‐promoting roles of activin A in vivo and in vitro.

## 2 Results

### 2.1 INHBA is Overexpressed in PDAC Cells and Correlates with Poor Survival Rates Among PDAC Patients

**Figure 1:**
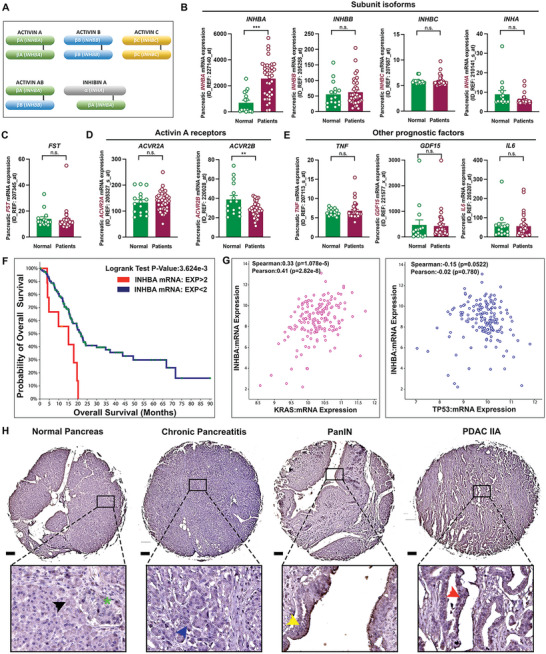
*INHBA* and activin A expression are overexpressed in pancreatic cancer. A) Structure of activins and inhibin A. B–E) Gene expression for inhibin subunit isoforms, *FST*, activin receptors, *TNF*, *GDF15*, and *IL6*; Outliers were removed using GraphPad(*α* = 0.05). F) Kaplan‐Meier overall survival plots by *INHBA* mRNA expression [(EXP>2, Red line) vs (EXP<2, Blue line)]. G) Correlation between *INHBA* and *KRAS* or *TP53* genes. H) IHC staining for inhibin *β*A; normal acinar cell (black arrow), endocrine cells (green asterisk), atrophic acinar cell (blue arrow), PanIN lesion (yellow arrow), and PDAC cell (red arrow). **P<0.01; ***P<0.001; n.s., not significant.

Inhibin *β*A is a subunit for activin isoforms (**Figure** **1**A). First, we characterized the expression of activins and inhibin A subunits in normal human pancreas and pancreatic tumors by accessing the Gene Expression Omnibus database using the accession number GSE16515. *INHBA* expression was significantly upregulated in tumors than in normal pancreas, but expressions for other isoforms, *INHBB* and *INHBC*, remained unchanged (Figure 1B). The expressions of *INHA* and *FST* genes were downregulated and statistically non‐significant, respectively (Figure 1B,C). Additionally, *ACVR2A* expression was similar between normal pancreas and tumor, whereas *ACVR2B* was downregulated in the tumors (Figure 1D). Further, we compared expressions for *TNF*, *GDF15*, and *IL6* which are recognized as prognostic cytokines for PDAC along with activin A.^[^ ^18^ ^]^ Unlike *INHBA*, expressions of *TNF*, *GDF15*, and *IL6* genes were not statistically significant between normal pancreas and tumors (Figure 1E).

Next, we examined the correlations between *INHBA* expression and PDAC survival rates or expression of oncogene *KRAS* and *TP53* in patients using The Cancer Genome Atlas‐Pancreatic Adenocarcinoma on cBioPortal (https://www.cbioportal.org). Pancreatic *INHBA* expression is correlated with poor PDAC survival (Figure 1F). *INHBA* expression was positively correlated with *KRAS* expression and showed a downward trend as *TP53* expression was increased in tumors (Figure 1G). Further, IHC analysis with TMA identified that the expression of inhibin *β*A protein was induced in atrophic acinar cells (blue arrow) and was intensively detected in PanIN lesions (yellow arrow) and PDAC cells (red arrow) (Figure 1H). Consistently, inhibin *β*A was significantly induced in atrophic acinar cells than in normal acinar cells and overexpressed in PDAC cells on two KPC tumors which we used in our previous publication (Figure S1, Supporting Information).^[^ ^11^ ^]^ Also, stromal cells express inhibin *β*A in human and mouse PDAC (Figure 1H & Figure S1, Supporting Information).

### 2.2 KPC8069 Exhibits the Highest Activin A Secretion as well as Activity of Chemoinvasion

**Figure 2:**
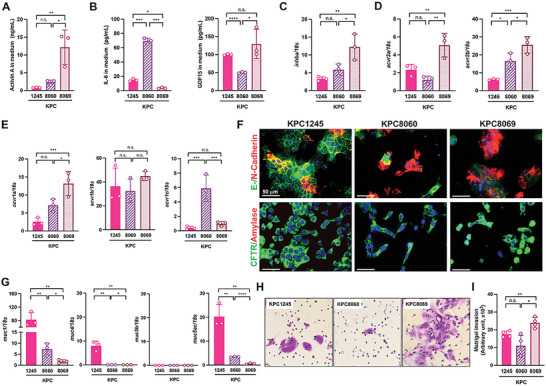
KPC8069 cells have high expressions of the *Inhba* gene and activin A and exhibit invasive potential. A,B) Medium Activin A, IL‐6, and GDF15 levels from different KPC cell lines (n = 3/line). C–E) RT‐PCR analysis of KPC cell lines(n = 3/line) with the reference of normal pancreas. F) Representative co‐immunofluorescence staining of E‐/N‐Cadherin and CFTR/Amylase(n = 3/line). Scale bar = 50 µm. G) RT‐PCR analysis of KPC cell lines(n = 3/line) with the reference of normal pancreas. H) Representative crystal violet staining of invaded cells on the basolateral side of transwell membrane(n = 4/line). Images were taken at 20x objective magnification. I) The stained area of each membrane is calculated by ImageJ and expressed as arbitrary units. *P<0.05; **P<0.01; ***P<0.001; ****P<0.0001; n.s., not significant.

Nearly 95% of PDAC tumors harbor *KRAS* mutations and 70% of PDAC tumors have *KRAS* and *TP53* mutations.^[^ ^1^ ^]^ Different cancer subtypes with different secretory states and the presence of non‐tumor cells such as fibroblasts and immune cells constitute intratumor heterogeneity of PDAC.^[^ ^14^, ^19^ ^]^ Here we compared mouse PDAC cell lines harboring *Kras^G12D^* and *Trp53^R172H^* mutations (KPC), KPC1245, 8060, and 8069, in the secretion of PDAC prognostic cytokines. KPC8069 had significantly higher secretion of activin A when compared to KPC1245 and 8060 (**Figure** **2**A). IL‐6 levels were the highest in the media from KPC8060 and lowest in KPC8069 (Figure 2B). GDF15 levels were statistically higher in the media from KPC8069 and 1245 than in KPC8060 (Figure 2B). Different gene expression levels for *Inhba* and activin receptors were observed across KPC cell lines. KPC8069 showed the highest expression levels of genes for *Inhba, Acvr2a, Acvr2b*, and *Acvr1a* (Figure 2C–E). *Acvr1b* expression was similar among KPC cell lines, but *Acvr1c* was the highest in KPC8060 (Figure 2E).

Mesenchymal N‐cadherin is implicated to induce stemness properties of cancer cells and metastasis through epithelial‐to‐mesenchymal transition and correlates with poor PDAC survival.^[^ ^20^ ^]^ Next, we examined the correlation between the expression of prognostic cytokines and invasion in KPC cell lines. Epithelial cadherin (E‐cadherin) protein was abundantly detected in KPC1245 but less in KPC8060 and 8069 (Figure 2F). Conversely, N‐cadherin was enriched in KPC8060 and 8069 and less detected in KPC1245 cells. All KPC cell lines were negative with amylase but positive with cystic fibrosis transmembrane conductance regulator (CFTR), a ductal cell marker (Figure 2F). Coincidently, the expression of genes for mucins (*Muc1*, *4*, and *5ac*), commonly referred to as malignant epithelial cell markers,^[^ ^21^ ^]^ was statistically higher in KPC1245, whereas KPC8069 had the lowest expression levels of the genes when normal pancreatic tissue was used as reference expression (Figure 2G). The *Muc5b* gene was significantly downregulated in all the KPC cell lines when compared to normal pancreatic tissue (Figure 2G). In the Matrigel invasion assay, KPC8069 exhibited faster invasion and migration than KPC1245 and 8060, but no statistical difference between KPC1245 and 8060 was observed (Figure 2H,I).

Finally, we screened the inhibitory effects of four different *Inhba* siRNAs in KPC8069 because of high activin A expression. All the tested *Inhba* siRNAs at 10 nm showed nearly 80% inhibition on the *Inhba* expression in KPC8069 (Figure S2A, Supporting Information). We pursued further testing and experiments with #4 *Inhba* siRNA. The *Inhba* siRNA consistently reduced *Inhba* expression by approximately 70% and 50% at 70 and 100% confluence, respectively (Figure S2B, Supporting Information). Activin A secretion was also hindered by the *Inhba* siRNA in KPC8069 cells (Figure S2C, Supporting Information).

### 2.3 Inhba siRNA Treatment Retards Orthotopic Tumor Growth/Metastasis and Improves Weight Loss and Survival

**Figure 3:**
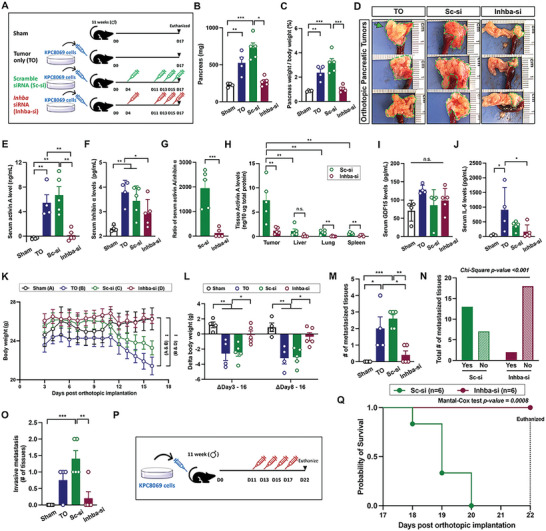
*Inhba* siRNA retards orthotopic tumor growth/metastasis and improves weight loss and survival in vivo. A) Experimental design. 12‐week‐old male mice were subject to surgery; all the mice received the active procedure for KPC8069 cell implantation into the pancreas except for the sham group which underwent the surgical procedures without cell implantation. The mice were randomly divided into groups and were intraperitoneally treated with designated treatments. B,C) Pancreas weight uncorrected and corrected for body weight (%). D) Representative gross appearance of tumors from orthotopic mice; green lines and arrows indicate tumors. E–H) Serum activin A, inhibin A, the ratio of activin A to inhibin A, and tissue activin A levels. I,J) Serum GDF15 and IL‐6 levels. K) Average daily body weights: *P*<0.01 for Sham and Sc‐si groups (A&C) and *P*<0.01 for Sc‐si and Inhba‐si groups (C, D). L) Body weight changes. M) Average number of metastasized tissues(per mouse). N) Chi‐square test for the total number of metastases between Sc‐si and Inhba‐si groups. O) An average number of metastasized tissues and histologically confirmed invasive metastasis. P) Survival test scheme; Orthotopic mice were randomly divided into two groups, were intraperitoneally treated with either scramble siRNA or *Inhba* siRNA every other day from D11 to D17, and were followed to record survival probabilities for groups. Q) Kaplan–Meier survival curve for the survival of Sc‐si and Inhba‐si groups with the Mantel‐Cox test. *P<0.05; **P<0.01; ***P<0.001; n.s., not significant.

Next, we examined whether suppressing tumor activin A would retard tumor growth by employing an orthotopic PDAC model. Detailed group information is available in Experimental Section (**Figure** **3**A). The siRNA was packaged with cholesterol‐modified polymeric CXCR4 inhibitor (PCX) for tumor‐targeted delivery during tumor growth.^[^ ^22^ ^]^ Because the knockdown efficiency of *Inhba* siRNA was influenced by cell confluence(Figure S2B, Supporting Information), we had the initial siRNA injection on day 4 (D4) of implantation and had three injections every other day from D11 as most of the pancreas became tumor at D12 as detected by ultrasound imaging (Figure S3, Supporting Information). As shown in Figure 3B,C, significant increases in the pancreas weight and pancreas weight corrected for body weight were observed in both TO and Sc‐si groups. Figure 3D shows the gross appearance of tumors formed in the groups. Serum activin A and inhibin A were considerably elevated in TO and Sc‐si groups when compared to the Sham group (Figure 3E,F). The ratio of activin A to inhibin A showed that circulating activin A was dominantly elevated in our model with a 2000‐fold increase in the Sc‐si group (Figure 3G), suggesting that the antagonistic role of inhibin A against activin A would be negligible. Furthermore, tissue activin A level was sevenfold higher in tumors than in other organs in the Sc‐si group (Figure 3H). However, no differences in serum GDF15 were found between groups, and serum IL‐6 was greatly increased in the TO group (Figure 3I,J). There was no statistical difference between TO and Sc‐si groups unless otherwise stated. Contrastingly, *Inhba* siRNA significantly retarded orthotopic tumor growth as demonstrated by smaller pancreas weights and tumor size in the gross images (Figure 3B–D). Serum activin A and inhibin A levels in the Inhba‐si group were similar to the Sham group (Figure 3E,F). The relative ratio of serum activin A to inhibin A and tumor activin A levels were remarkably reduced when compared to the Sc‐si group (Figure 3G,H). Serum IL‐6 levels were also significantly reduced in the Inhba‐si group than in the TO group.

The average body weight started to decline from D12 in both TO and Sc‐si groups. Until D16, both TO and Sc‐si groups lost 3.5 g on average, which largely happened between D8 and D16 (Figure 3K,L). However, *Inhba* siRNA prevented weight loss (Figure 3K,L). Furthermore, significant size reductions in epididymal adipocytes and quadriceps skeletal muscle fiber were mitigated by *Inhba* siRNA treatment (Figure S4A,B, Supporting Information).

Reduced tumor size by *Inhba* siRNA correlated with less metastasis. A post‐mortem examination was performed to identify small nodules on the tissue surface and to test tissue hardness on four nearby tissues: the spleen, liver, kidney, and intestinal lumen (Figure S5A, Supporting Information). We found nodules on 8 out of 16 tissues from the TO group (n = 4), 13 out of 20 tissues from the Sc‐si group (n = 5), and 2 out of 20 tissues from the Inhba‐si group (n = 5) (Figure 3M and Table S1, Supporting Information). A chi‐square test between groups revealed that there is no statistical difference between TO and Sc‐si groups, whereas the Inhba‐si group had a significantly reduced number of metastasized tissues when compared to TO and Sc‐si groups (Figure 3N and Table S1, Supporting Information). Furthermore, we investigated how many tissues were invasively metastasized with H& staining (Figure 3O and Figure S5A, Supporting Information). TO group had invasive metastasis in 100% of the livers (Table S2, Supporting Information). The Sc‐si group also had metastasis in 100% of the livers, 33% of the kidneys, and 100% of the intestine. However, the Inhba‐si group had invasive metastasis in only 40% of the livers. Invasive metastasis is indicated by a green asterisk and blue arrows for metastasis direction (Figure S5A, Supporting Information). We confirmed metastasized cancer cells in the liver through IHC for CK19 (Figure S5B, Supporting Information).

We next questioned whether *inhba* knockdown increases survival rates in our model. To avoid tumor size‐related contribution to survival rates, *inhba* siRNA was injected every other day from D11 to D17 (Figure 3P), given that most of the pancreas became tumors at D11 in our previous set of experiments (Figure 3B). In the Sc‐si group, all animals died by D20, whereas no death was observed in the Inhba‐si group until D22 (Figure 3Q). The average body weight of the Sc‐si group was dramatically decreased, and the mice in the Inhba‐si group maintained their body weights until D22 (Figure S5C, Supporting Information) with no changes in food intake observed (Figure S5D, Supporting Information). Weight loss in our model would be mediated independently of food intake.

### 2.4 Tumor‐Targeted INHBA Knockdown Restrains KPC Cancer Cells and Results in Less Fibrosis in the Pancreas

**Figure 4:**
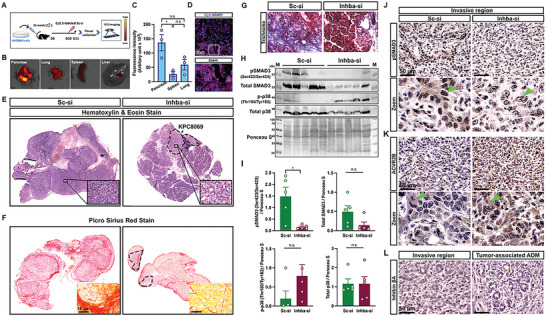
Tumor activin A suppression prevents cancer invasion and fibrosis accumulation through SMAD3 inactivation. A) Experimental design for PCX biodistribution (n = 3); 12‐week‐old orthotopic mice were intraperitoneally injected with Cy5.5‐labeled scramble siRNA at D10. After 24 h, tumors and tissues were harvested, and Cy5.5 tissue accumulation was evaluated under IVIS. B) Representative image of Cy5.5 fluorescence. C) Fluorescence intensities were measured by ImageJ and compared. D) Representative Cy5.5 and DAPI fluorescence images of frozen‐sectioned tumor tissue. Scale bar = 50 µm. E,F) Representative H&E and PSR staining of tumor sections from Sc‐si and Inhba‐si groups. Black‐dotted circles for KPC8069 tumor cells in Inhba‐si groups. G) Representative Trichrome staining of tumor sections (n = 5/group). H) Western blot bands. M, marker. I) Relative protein expression that is normalized by ponceau S staining. J–L) Representative DAB staining for pSMAD3, ACVR2B, and inhibin *β*A of tumor sections (n = 5/group). Green arrows indicate the signals of each protein. Scale bar = 50 µm. *P<0.05; n.s., not significant.

The tumor‐targeted siRNA delivery of PCX has been characterized previously.^[^ ^22^ ^]^ We confirmed PCX biodistribution in our model by tagging PCX with Cy5.5‐labeled scramble siRNA (**Figure** **4**A). After 24 h of injection, we imaged the accumulation of Cy5.5 fluorescence in different tissues such as the heart, lung, pancreatic tumor, kidney, liver, spleen, skeletal muscles, and adipose tissues (Figure S6, Supporting Information). Cy5.5 fluorescence confirmed abundant accumulation in tumor tissues, followed by the lung and spleen (Figure 4B,C). Confocal imaging further showed that Cy5.5‐labeled siRNA was distributed on the entire surface of frozen tumor sections (Figure 4D).

The analysis of tumor histology was performed to examine the impact of activin A suppression on PDAC tumors. H&E showed that tumors from the Sc‐si group exhibit complete loss of acinar cells but the presence of abnormal cells (Figure 4E). A significant accumulation of fibrosis was detected (Figure 4F). Masson's Trichrome staining identified the significant accumulation of collagen type 1 in the tumors from the Sc‐si group (Figure 4G). The local invasion of cancer cells and accumulation of fibroblasts was confirmed through IHC staining of CK19 as a cancer cell marker and *α*‐SMA as an activated fibroblast marker (Figure S7A, Supporting Information). In addition, we found the occurrence of tumor‐associated acinar‐to‐ductal metaplasia (ADM) and a significant accumulation of *α*‐SMA‐positive fibroblasts around the tumor‐associated ADM regions (Figure S7B, Supporting Information). However, *Inhba* siRNA restrained the local invasion of cancer cells, and the majority of the sections from the Inhba‐si group preserved acinar cells (Figure 4E and Figure S7A, Supporting Information). Although *α*‐SMA‐positive fibroblasts were detected around CK19‐positive cells (Figure S7A, Supporting Information), less fibrosis accumulation and collagen type 1 deposition were observed in the tissue sections (Figure 4F,G). Also, we found less accumulation of *α*‐SMA‐positive fibroblasts near tumor‐associated ADM regions from the Inhba‐si group (Figure S7B, Supporting Information).

Tumor SMAD3 correlated with poor PDAC outcomes such as tumor size and metastasis in PDAC patients.^[^ ^13^ ^]^ Activin A signals through canonical SMAD3‐dependent pathways and/or non‐canonical pathways which involves p38 phosphorylation.^[^ ^8^ ^]^ To understand the potential signaling pathway of activin A in PDAC, we analyzed the phosphorylation status of tumor SMAD3 and p38 proteins (Figure 4H). *Inhba* siRNA significantly reduced SMAD3 phosphorylation but p38 phosphorylation was statistically not‐significant when compared to the Sc‐si group (Figure 4I), suggesting that activin A might act through SMAD3 phosphorylation during orthotopic tumor growth. Next, IHC for pSMAD3, ACVR2A, and ACVR2B was performed to identify target cells of activin A in tumors. pSMAD3 signal was detected in the nuclei of most cells in the tumors from Sc‐si (Figure 4J). Furthermore, ACVR2A and ACVR2B staining showed a consistent pattern with pSMAD3, but the ACVR2A signal was weaker than ACVR2B (Figure 4K and Figure S7C, Supporting Information). These findings indicate that activin A targets both cancer cells and CAFs in our model. Additionally, we observed high expression of pSMAD3 and ACVR2B in tumor‐associated ADM regions (Figure S7D, Supporting Information), suggesting a potential role of activin A in tumor‐mediated ADM.

In addition, the activin A source was determined through IHC staining with the inhibin *β*A antibody on the tumor section. Inhibin *β*A was intensely detected in cancer cells and fibroblasts (Figure 4L, left). In addition, high expression of inhibin *β*A was detected in tumor‐associated ADM region (Figure 4L, right). Therefore, tumor and systemic activin A levels would originate from cancer cells, fibroblasts, and ADM as consistent with IHC findings in TMAs (Figure 1H).

### 2.5 Activin A Promotes KPC Tumor Cell Proliferation through SMAD3 Signaling

**Figure 5:**
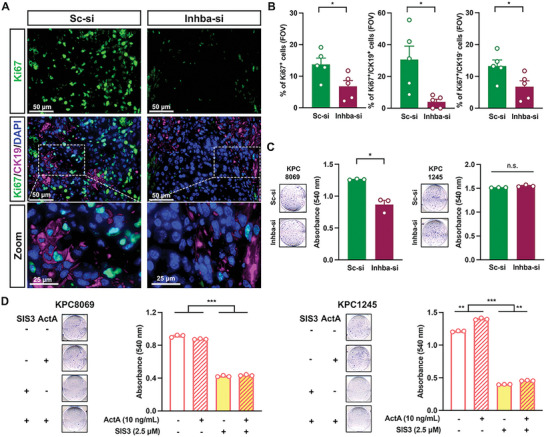
*Inhba* siRNA decreased the number of proliferating cells in tumors and the proliferation of KPC8069 cells. A) Representative IF staining of tumors with Ki67 and CK19(n = 5/group). Scale bar = 50 µm. B) The number of Ki67‐positive cells among CK19‐positive, negative, or both cells from the field of view, FOV (n = 5/group). C,D) Representative cell staining with 0.1% crystal violet, and absorbances of KPC8069 (left) and KPC1245 (right) with exogenous activin A and/or SIS3. *P<0.05; **P<0.01; ***P<0.001; n.s., not significant.

Next, we investigated if *Inhba* knockdown decreases cancer proliferation which would mediate stalled tumor growth in vivo. IF staining showed that Ki67‐positive cells were abundantly detected in the tumors from the Sc‐si group (**Figure** **5**A). Co‐staining with CK19 revealed that Ki67 was detected in CK19‐positive and CK19‐negative cells in the tumors from the Sc‐si group (Figure 5A). However, Ki67 was barely detected in the tumors from the Inhba‐si group (Figure 5A). We quantified the total number of Ki67‐positive cells and the number of Ki67‐positive cells among CK19‐positive or negative cells (Figure 5B). When compared to the Sc‐si group, the Inhba‐si group had a significantly reduced total number of Ki67‐positive cells and number of Ki67‐positive cells among CK19‐positive cells in the tumors (Figure 5B). The number of Ki67‐positive cells among CK19‐negative cells was also significantly lower in the Inhba‐si group. These data indicate that activin A upregulates the proliferation of cancer and cancer‐associated cells in PDAC tumors.

We further investigated whether *Inhba* siRNA directly suppresses the proliferation of PDAC tumor cells using KPC8069 and KPC1245 which showed the highest and lowest activin A secretion (Figure 2A). When compared to the cells treated with scramble siRNA, *Inhba* siRNA significantly decreased the proliferation of KPC8069 but showed no inhibitory effects on KPC1245 proliferation (Figure 5C). Inversely, exogenous activin A had no effects on KPC8069 proliferation but significantly enhanced KPC1245 proliferation (Figure 5D). SIS3, a selective inhibitor of SMAD3 phosphorylation, greatly decreased the proliferation of both KPC8069 and KPC1245 in the presence or absence of exogenous activin A (Figure 5D). These findings implicate that *Inhba* siRNA directly suppresses the proliferation of a subset of *Inhba*‐high KPC cells that reciprocally promote the proliferation of *Inhba*‐low tumor cells.

### 2.6 Inhba Knockdown Increases Tumor *α*‐SMA Expression, T‐Cell Infiltration, and BAX Expression in Tumor Cells

**Figure 6:**
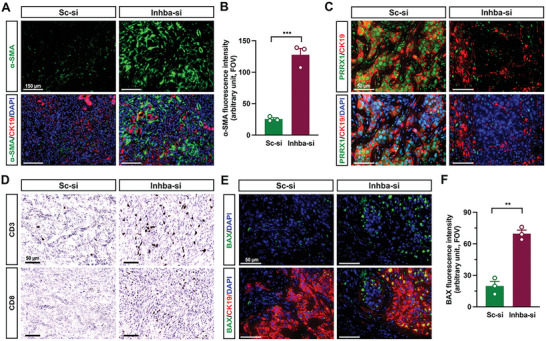
*Inhba* siRNA increases the accumulation of *α*‐SMA^high^ fibroblasts and infiltration of cytotoxic T cells. A) Representative IF staining with *α*‐SMA and CK19 of tumor sections(n = 3/group). Scale bar = 150 µm. B) Mean fluorescence intensity of *α*‐SMA. Field of view(FOV; n = 3/group). C) Representative IF staining with PRRX1 and CK19 of tumor sections(n = 3/group). Scale bar = 50 µm (B). D) Representative DAB staining of CD3 and CD8 with tumor sections(n = 3/group). scale bar = 50 µm. E) Representative IF staining of BAX and CK19(n = 3/group). Scale bar = 50 µm. F) Fluorescence intensity of BAX. **P<0.01; ***P<0.001.

Because we observed SMAD3 phosphorylation in stromal cells (Figure 4J), we further extended our question to whether *Inhba* knockdown influences CAF activation states and subsequent T cell infiltration which would be an alternative pathway to hinder tumor growth and metastasis (Figure 3). Interestingly, tumor fibroblasts from the Sc‐si group had a weak expression of *α*‐SMA (**Figure** **6**A). However, *α*‐SMA was intensely and abundantly found in the cells especially surrounding CK19‐positive cells in the tumors from the Inhba‐si group (Figure 6A). Quantification of *α*‐SMA fluorescence indicates considerably high expression of *α*‐SMA in the Inhba‐si group (Figure 6A,B). The knockout of *Prrx1* promotes the accumulation of *α*‐SMA^high^ fibroblasts.^[^ ^16b^ ^]^ PRRX1 was abundantly expressed in the nuclei of CK19‐positive and CK19‐negative cells in tumors from the Sc‐si group, whereas PRRX1 was weakly expressed, and the majority of weak PRRX1 signaling was detected in CK19‐positive cells in tumors from Inhba‐si group (Figure 6C). In addition, increased infiltration of *α*‐SMA^high^ fibroblasts correlated with high infiltration of T cells. Pan T cells (CD3‐positive) and cytotoxic T cells (CD8‐positive) were abundantly detected in the tumor beds from the Inhba‐si group, but those cell types were almost absent in the tumor beds from the Sc‐si group (Figure 6D). Furthermore, apoptotic BAX expression was examined in tumor cells. Greatly increased BAX expression in CK19‐positive cells was detected in the Inhba‐si group when compared to in Sc‐si group (Figure 6E). The intensity of BAX in CK19‐positive cells was statistically higher in the Inhba‐si group than the Sc‐si group (Figure 6E,F). However, PD‐L1 expression in CK19 cells remained stable in the Inhba‐si group when compared to the Sc‐si group (Figure S8, Supporting Information). These observations suggest that activin A suppression promoted the accumulation of *α*‐SMA^high^ fibroblasts and increased cytotoxic T‐cell infiltration and cancer apoptosis.

### 2.7 Activin A Decreases *α*‐SMA but Increases IL‐6 in Pancreatic Fibroblasts to Promote KPC8069 Growth

**Figure 7:**
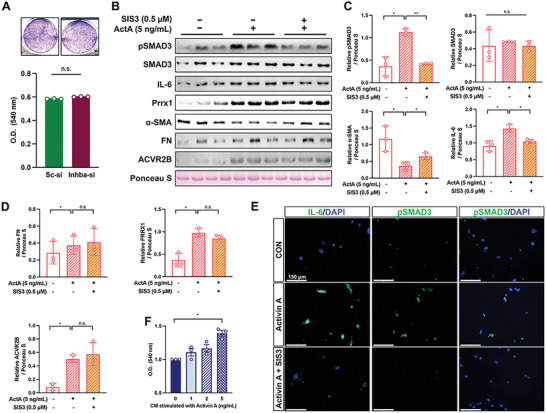
Activin A downregulates *α*‐SMA and upregulates IL‐6 in PSCs to promote KPC proliferation. A) PSC proliferation by *Inhba* siRNA. B) Western blot analysis of PSCs stimulated with activin A (5 ng mL^−1^) in the absence or presence of SIS3 (0.5 µm). C,D) Relative protein expression after normalization with ponceau S staining. E) Representative immunocytochemistry staining of IL‐6 or pSMAD3 with PSCs. Scale bar = 150 µm. F) Absorbance of crystal violet from KPC 8069 24 h after the treatment with conditioned media from PSCs. In vitro data were obtained from three independent experiments(n = 3). *P<0.05; **P<0.01; n.s., not significant.

Because CAF activation status was skewed towards *α*‐SMA^high^ fibroblasts, and fewer fibroblasts were accumulated in tumors by activin A suppression, we examined the role of activin A in the proliferation and activation of PSC. Although *Inhba* siRNA did not affect the proliferation of primary mouse PSC (**Figure** **7**A), activin A significantly increased the expression levels of ACVR2B and SMAD3 phosphorylation and coincidently increased the expression levels of IL‐6, PRRX1, and fibronectin (FN) but decreased *α*‐SMA levels (Figure 7B,C). In the presence of SIS3, activin A failed to induce SMAD3 phosphorylation, upregulation of IL‐6, and suppression of *α*‐SMA. The expression of FN, PRRX1, and ACVR2B remained unchanged after being upregulated by activin A in the presence of SIS3 (Figure 7B,D). We confirmed the upregulation of IL‐6 and SMAD3 phosphorylation by activin A through the SMAD3 pathway using IF staining (Figure 7E).

Further, we examined whether CM from PSC stimulated with activin A would promote KPC8069 proliferation. We observed that the CM from PSC stimulated with activin A at 5 ng mL^−1^ significantly promoted KPC8069 proliferation (Figure 7F). Collectively, the in vitro observation indicates that activin A indirectly induces tumor proliferation through fibroblasts.

Abundant staining of pSMAD3 and ACVR2B in tumor‐associated ADM extended our question to the role of activin A in tumor‐associated ADM (Figure S7D, Supporting Information). As consistent with in vivo observations, activin A promoted phosphorylation of SMAD3 and expression of SOX9, a marker for ADM, in acinar 266‐6 cells (Figure S9, Supporting Information). However, it is unclear if tumor‐associated ADM becomes cancerous but will constitute PDAC tumor in part by secreting activin A and promoting the accumulation of fibroblasts as demonstrated by our study (Figure S7B, Supporting Information, and Figure 4L) and human invasive PDAC.^[^ ^23^ ^]^

## 3 Discussion

Activin A has been recognized as a prognostic factor for PDAC.^[^ ^12^ ^]^ Here, we demonstrated that gene expression of *INHBA*, not other subunit isoforms, was significantly upregulated in pancreatic tumors than in normal pancreatic tissues through secondary analysis of publicly accessible gene expression data. Regarding a source cell of activin A, Mancinelli et al.^[^ ^12a^ ^]^ reported that the inhibin *β*A subunit is detected in acinar, stromal, and tumor cells of human pancreatic tumors in TMA. In support of the previous observation, our DAB staining with TMA indicates that the inhibin *β*A subunit was transiently overexpressed from atrophic acinar cells in chronic pancreatitis to the cells in PanIN, and was highly overexpressed in human PDAC. Consistent observations were made in KPC tumors and our orthotopic mice.^[^ ^24^ ^]^ Moreover, tissue activin A levels were nearly 7 times higher in tumors than in other tissues. Thus, we suggest tumor and stromal cells as source cells of local and systemic activin A in PDAC.

Additionally, activin A would be a potential therapeutic target for PDAC beyond its recognition as a prognostic factor for PDAC. To the best of our knowledge, we first report that activin A suppression, which is acquired through tumor‐targeted *Inhba* siRNA delivery, retarded orthotopic tumor growth/metastasis and improved weight loss and survival. In vitro data indicate that activin A promotes KPC cell proliferation through SMAD3 phosphorylation. Consistently, Togashi, et al. demonstrated that activin A promoted MIA‐PaCa2 cell proliferation, however, *INHBA* overexpression showed no effects on heterotopic tumor growth in immunodeficient mice.^[^ ^25^ ^]^ The null effects on tumor size by *INHBA* overexpression would be related to the model used,^[^ ^25^ ^]^ including subcutaneous cell implantation, lack of metastasis, and T‐cell deficiency, all of which would potentially modulate tumor growth.^[^ ^26^ ^]^ Furthermore, Zhong, et al. also reported that the inhibitory effects of soluble ACVR2B/Fc on orthotopic tumor growth were not statistically significant, although a 30% reduction in tumor weight was observed.^[^ ^12b^ ^]^ The borderline significance would pertain to the use of KPC cells with low *Inhba* expression for generating an orthotopic model which elevates plasma activin A level close to 1.5 ng mL^−1^.^[^ ^12b^ ^]^ Contrarily, our model achieved activin A level of above 5 ng mL^−1^ which is within the range of activin A detected in PDAC patients.^[^ ^11^ ^]^ Furthermore, we observed that *Inhba* siRNA did not have effects on KPC1245 proliferation which expresses a relatively low *Inhba* gene, whereas *Inhba* siRNA suppressed the proliferation of KPC8069 which showed a relatively high *Inhba* expression.

We found that activin A modulates the activation state of CAFs in PDAC. Microenvironmental heterogeneity is a risk factor for metastasis and survival in PDAC patients and is related to the activation status of CAFs.^[^ ^27^ ^]^ MyoCAFs show relatively higher expression levels of myofibroblast genes such as *Acta2* for *α*‐SMA. iCAFs express relatively higher levels of inflammatory genes such as Il‐6. Both types are shown to be interconvertible as demonstrated by Ohlund, et al.^[^ ^28^ ^]^ In PDAC patients, a low histopathological score for *α*‐SMA predicted poorer survival in PDAC patients.^[^ ^16a^ ^]^ In PDAC mice, the deletion of *Acta2*‐expressing CAFs significantly decreased survival.^[^ ^16a^ ^]^ Conversely, overexpression of fibroblast *α*‐SMA by *Prrx1* knockout prevented PDAC metastasis in orthotopic PDAC mice.^[^ ^16b^ ^]^ Furthermore, the tumor beds enriched with *α*‐SMA^high^ CAFs resulted in high infiltration of immune cells such as CD3‐positive or CD8‐positive T cells.^[^ ^16b^ ^]^ Therefore, a body of evidence highlights the importance of CAF activation status in association with tumor growth and metastasis. However, little is known regarding the potential regulators of CAF activation in PDAC. In our model, tumor activin A suppression retarded tumor growth and metastasis. Activin A suppression increased the accumulation of *α*‐SMA^high^/PRRX1^low^ CAFs and led to the infiltration of CD3‐ or CD8‐positive T cells with a concomitant increase of BAX expression in PDAC tumor cells. In in vitro data, we confirmed that activin A increases the protein expression of IL‐6 and PRRX1 but decreases *α*‐SMA protein expression in PSCs. We suggest SMAD3 activation as a responsible target pathway for regulating IL‐6 and *α*‐SMA expression by activin A. Activin A‐mediated PRRX1 upregulation seems to be mildly inhibited by SMAD3 inhibitor but requires further characterizations for potential mechanisms behind it.

There is a body of evidence implicating the tumor‐suppressing roles of activin A in PDAC. The ablation of the *Acvr1b* gene promoted *Kras^G12D^*‐mediated tumorigenesis and decreased PDAC survival in mice.^[^ ^29^ ^]^ Additionally, consistent observations were made by the loss of the *Smad4* gene, in mice harboring *Kras* ^G12D.[^ ^30^ ^]^ However, loss of *Acvr1b* or *Smad4* occurs before *Kras^G12D^*‐dependent tumor development.^[^ ^29^, ^30^ ^]^ Therefore, the evidence addresses the importance of activin A signaling in tumorigenesis, especially the development of PDAC precursor lesions. Accordingly, the previous reports^[^ ^29^, ^30^ ^]^ demonstrated that the loss of activin A signaling accelerates the occurrence rates of premalignant intraductal papillary mucinous neoplasia (IPMN), whereas *Kras^G12D^* only elicits PanIN, another precursor lesion. Consistently, the treatment of soluble ACVR2B/Fc at 1.5 months old age, before PDAC develops, promoted the development of cystic lesions, which are reminiscent of a high incidence of IPMN by loss of activin A signaling during *Kras^G12D^*‐dependent tumorigenesis.^[^ ^24^, ^29^, ^30^, ^31^ ^]^ Therefore, the consistent observations identify activin A as a risk factor predisposing to the development of IPMN when *Kras^G12D^* is present.

## 4 Conclusion

We demonstrate that activin A suppression renders favorable outcomes for PDAC including the mitigation of tumor growth/metastasis and weight wasting and improvement of survival in mice. As potential mechanisms, we suggest that activin A promotes tumor cell proliferation and suppresses *α*‐SMA expression in fibroblasts which coincidently promotes PDAC growth/metastasis and inhibits T cell infiltration. In vitro data indicate that activin A plays its tumor‐promoting roles in a SMAD3‐dependent manner. Therefore, current findings serve as a preclinical basis for targeting activin A expression in PDAC as a potential therapeutic avenue.

## 5 Experimental Section

### Cell Culture

Acinar 266‐6 (ATCC, CRL‐2151), KPC1245, 8060, and 8069 cell lines (gifts from Dr. Pankaj K. Singh and Dr. Michel A. Hollingsworth, UNMC) were cultured in RPMI1640 supplemented with 10% fetal bovine serum and 1% Antibiotic‐Antimycotic under a 5% CO_2_ incubator at 37 °C. All cell lines were used at the passages between 8 to 13. Conditioned media were collected from KPC8069 (KPC‐CM) at 100% confluence and filtered with a 0.02 µm pore size.

### Pancreatic Stellate Cell (PSC) Isolation and Culture

The isolation and culture of PSCs were performed by following Feldmann et al.^[^ ^16b^ ^]^ Briefly, normal mouse pancreatic tissues (n = 5) were minced and digested in Gey's balanced salt solution (GBSS) containing 0.3% bovine serum albumin, 0.05% collagenase P solution, 0.02% pronase, and 0.1% DNase. After centrifugation, the pellet was resuspended in 4.75 mL GBSS/0.3% BSA and 4 mL 28.7% Histodenz (Sigma, D2158) in GBSS solution. After adding a layer of 3 mL GBSS/0.3% BSA, the mixture was centrifuged, and the white layer above the interface was collected and cultured in the media of 50% DMEM‐F12 and 50% DMEM supplemented with 20% FBS and 1% antibiotic‐antimycotics. To collect the conditioned media (CM) from the cells, stellate cells were stimulated with activin A for 24 h, and then fresh media were added after washing twice with PBS and collected as CM after additional 24 h.

### Cell Growth and Invasion Assay

Cell growth experiments were performed by seeding cells at 3000 cells in a 6‐well plate in the presence or absence of designated treatments. After 48–72 h, the cells were fixed with 10% formaldehyde, washed with PBS three times, and stained with 0.1% crystal violet in PBS for 10 min. After washing with distilled water, the wells were air‐dried and eluted using 50% DMSO in PBS, and the absorbance was measured at 540 nm. Chemoinvasion activity was examined using 24‐well plates with transwell inserts coated with Matrigel (Corning, 354480). 5 × 10^5^ cells were seeded on the transwell inserts in 1% FBS‐RPMI, and the inserts were placed in 24‐well plates with 10% FBS‐RPMI. After 24 h, the invaded cells were fixed and stained with 0.1% crystal violet. Noninvaded cells were scraped off from the wells. Relative invasion abilities were compared by calculating cell‐invaded areas using ImageJ (NIH software, Fiji v2.3.1).

### Real‐Time (RT) PCR

Total RNAs were collected using TRIzol reagent and synthesized into cDNAs with the high‐capacity RNA‐to‐cDNA kit. RT‐PCR was performed on the CFX96 Real‐Time PCR Detection System (Bio‐Rad Laboratories, 185–5096). The target genes were normalized with *18s*, and the relative expression was calculated using the 2^−ΔΔCT^. The primers used are listed in Table S3, Supporting Information.

### Animal and Orthotopic Implantation

All procedures involving the use of mice were approved by the Institutional Animal Care and Use Committee (IACUC) at UNMC (19‐094‐9). The animals were given free access to food and water and kept in Comparative Medicine facilities.

12‐week‐old C57BL/6 male mice were used to generate an orthotopic PDAC model by implanting 5 × 10^4^ KPC8069 cells. The sham group received the same operation of procedure except for cell implantation. Postoperative monitoring was carried out for 3 days (D3), and the orthotopic mice were divided into 3 groups on D4: treatment with no siRNA (tumor only, TO), scramble siRNA (Sc‐si), or *Inhba* siRNA (Inhba‐si). Body weight and food intake were measured daily.

### Administration of siRNA‐PCX Nanoparticles

siRNA‐PCX nanoparticles were prepared as previously described.^[^ ^22^ ^]^ Polyplexes (2.5 mg kg^−1^ siRNA, 5 mg kg^−1^ PCX) were intraperitoneally injected into the orthotopic mice on D4, 11, 13, and 15. The sequences for the scramble and *Inhba* siRNA are listed in Table S3, Supporting Information. For biodistribution, Cy5.5‐labeled siRNA was mixed with PCX polyplexes and given to the orthotopic mice at D11. After 24 h, tissues were harvested for ex vivo fluorescence imaging using Xenogen IVIS 200 (675 or 720 nm). Tumors were encapsulated in Tissue‐Tek O.C.T. Compound, sectioned using cryostat at 8 µm, and stained with DAPI. In situ distribution of Cy5.5 fluorescence was imaged with the confocal microscope.

### Human Pancreatic Tissue Microarray

PDAC tissue microarrays (TMA) (US Biomax, Inc.) consisted of 60 cases of PDAC and 9 normal cases (3/case). Pancreatitis TMA had 14 cases of PanIN, 6 pancreatitis, and 4 PDAC (2/case). TMA slides were used for DAB staining.

### Evaluation of Serum Cytokines

Serum levels of activin A, inhibin A, IL‐6, and GDF15 were quantified using commercially available ELISA kits [Actinvi A and inhibin A ELISA kits (Ansh Labs, AL‐110 and AL‐123), IL‐6 ELISA (Invitrogen, BMS603‐2), and GDF15 Quantikine ELISA (R&D Systems, MGD150)].

### Immunohistochemistry (IHC) Assay

Tissue slides with 5 µm thickness were subject to hematoxylin/eosin (H&E), Masson's trichrome staining, or picrosirius red solution (PSR) for histological examination. DAB (3, 3′‐diaminobenzidine) and immunofluorescence assays were previously described.^[^ ^11^ ^]^ All the images were taken with the EVOS M7000 Imaging System (Invitrogen, AMF700). Antibody specificity was tested with positive control tissues (Figure S10, Supporting Information) and antibody information is listed in Table S4, Supporting Information.

### Immunoblotting

Tissue lysates were prepared using RIPA buffer supplemented with protease and phosphatase inhibitors. Proteins (20 µg) were separated on 12.5% SDS polyacrylamide gel, transferred to PVDF membranes, and incubated with primary and secondary antibodies. The primary antibodies used are listed in Table S4, Supporting Information. Protein bands were captured by a c500 imaging system (Azure Biosystems). The band intensities were determined using ImageJ software.

### Statistics

Graphs were generated by GraphPad Prism 9.3.1 software, and in vivo data were presented as mean±standard error of the mean (SEM). The one‐way analysis of variance (ANOVA) with Tukey's post‐hoc test was performed followed by unpaired two‐tailed Student's t‐tests. The number of each group for animal experiments started with 5/group except for the sham group (n = 4). During the experimental period, one from the TO group died on D15. Then, the sample size was justified using G*power software v3.1 with post‐hoc analysis using pancreas weights. The calculated power was 0.99. In vitro data were presented as mean±standard deviation (SD), and statistical significance was determined by paired t‐test. *P*‐value was expressed as follows; *P<0.05; **P<0.01; ***P<0.001; ****P<0.0001; n.s., not significant.

## Conflict of Interest

The authors declare no conflict of interest.

## Author Contributions

S.Y.Y., Ph.D. (Conceptualization: Lead, Data curation: Lead, Formal analysis: Lead, Investigation: Lead, Methodology: Lead, Project administration: Lead, Visualization: Lead, Writing – original draft: Lead, Writing – review & editing: Lead); Y.L., Ph.D. (Investigation: Supporting, Writing – review & editing: Supporting); S.T., Ph.D. (Conceptualization: Supporting, Investigation: Supporting, Writing – review & editing: Supporting); A.A., Ph.D. (Writing – review & editing: Supporting); R.D. (Writing – review & editing: Supporting); D.O., Ph.D. (Conceptualization: Equal, Resources: Equal, Writing – review & editing: Supporting); T.C.C., Ph.D. (Writing – review & editing: Supporting); M.A.H., Ph.D. (Writing – review & editing: Supporting); S.Y.K., Ph.D. (Conceptualization: Equal, Data curation: Lead, Funding acquisition: Lead, Investigation: Equal, Project administration: Equal, Resources: Lead, Supervision: Lead, Writing – original draft: Equal, Writing – review & editing: Equal).

## Supporting information

Supporting Information

## Data Availability

The data that support the findings of this study are available from the corresponding author upon reasonable request.
