# Supplementary material for: Uncovering Tumor‐Promoting Roles of Activin A in Pancreatic Ductal Adenocarcinoma
Source: Adv Sci (Weinh). 2023 Apr 21;10(16):2207010. doi: 10.1002/advs.202207010 (PMC10238186; doi:10.1002/advs.202207010)
Supplement: Supplementary file 1 — Supporting Information [file ADVS-10-2207010-s001.pdf]

ADVANCED SCIENCE
Open Access

Supporting Information

for *Adv. Sci.*, DOI 10.1002/advs.202207010

Uncovering Tumor-Promoting Roles of Activin A in Pancreatic Ductal Adenocarcinoma

*Seok-Yeong Yu, Yi Luan, Siyuan Tang, Amirhossein Abazarikia, Rosemary Dong, Thomas C. Caffrey, Michael A. Hollingsworth, David Oupicky and So-Youn Kim**

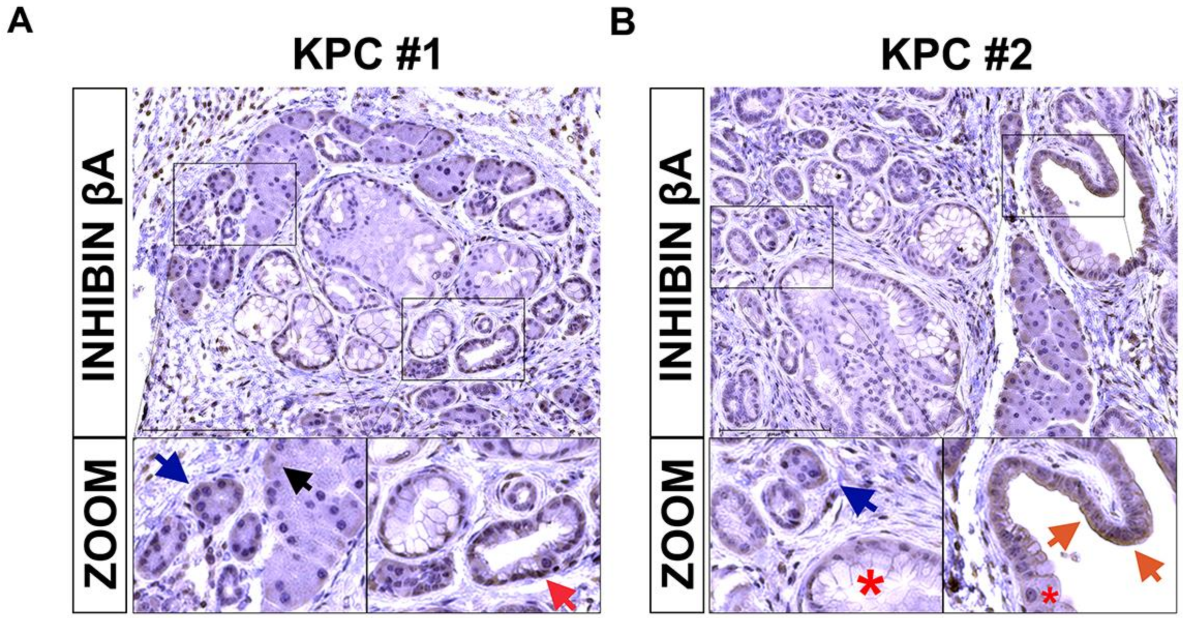

Supplemental figures

Figure S1. IHC of Inhibin βA on tumors from KPC mice. The black arrow is for acinar cells, the blue arrow for atrophic acinar cells, and the red arrow for PanIN in KPC #1. Red asterisk for PanIN and orange arrow for PDAC cells in KPC mouse #2.

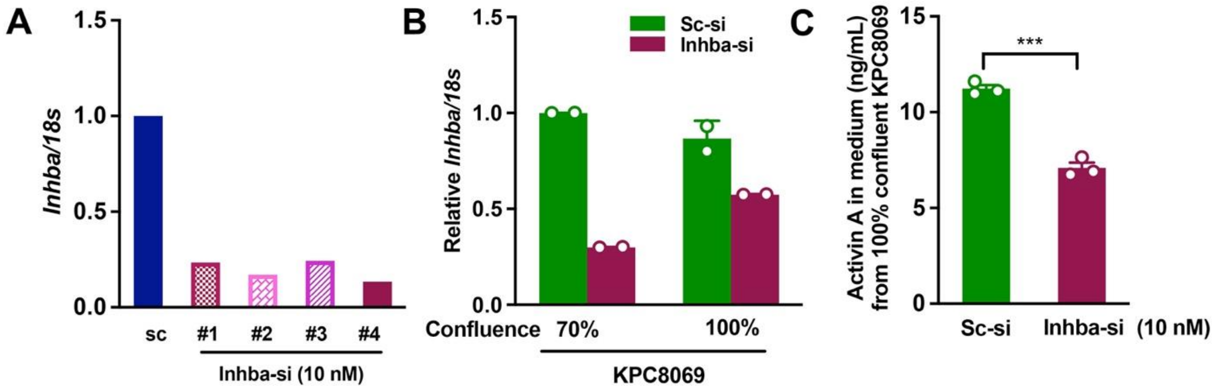

Figure S2. The inhibitory effects of Inhba-si in KPC8069 cells. A. Screening of inhibitory effects of four different *Inhba* siRNAs in KPC8069 cells. **B.** KPC8069 cells were treated with either scramble (Sc-si) or Inhba siRNA (Inhba-si) at 10 nM for 48 hours. The gene expression of Inhba was normalized with 18s. **C.** Activin A levels in media were measured using ELISA kit after 48 hour treatment of designated siRNA.

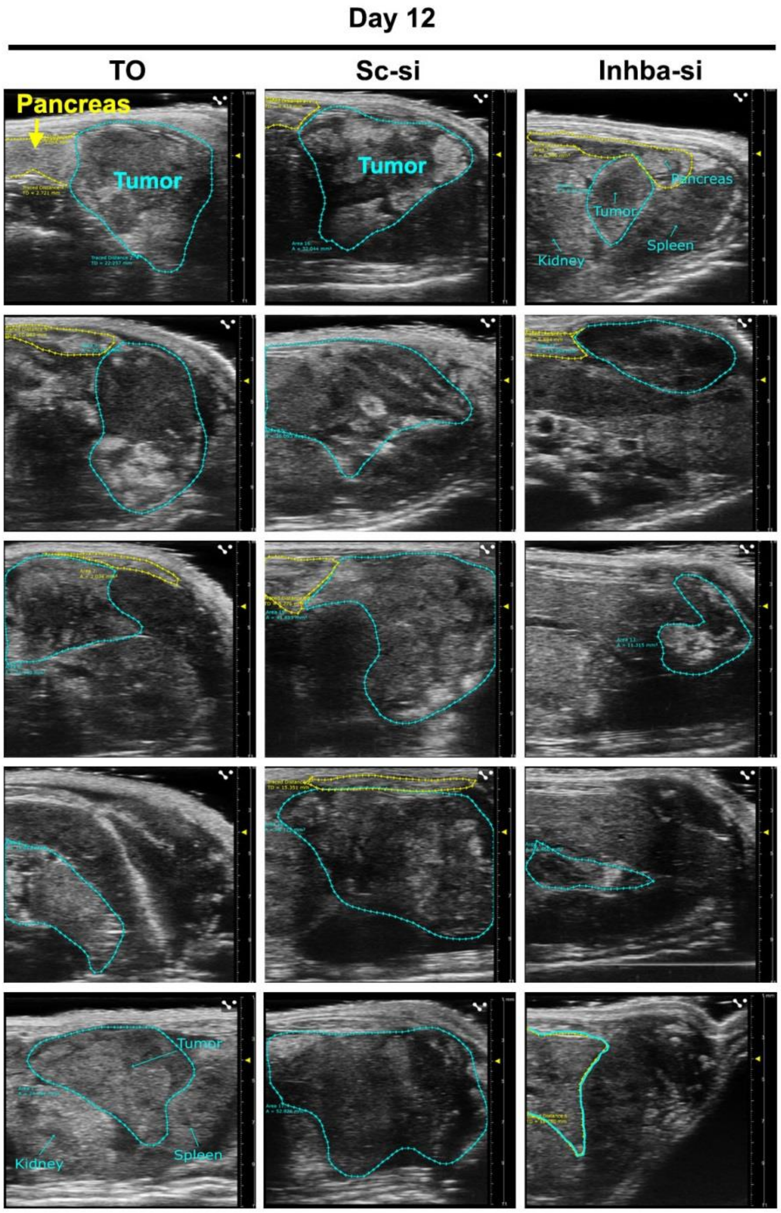

Figure S3. Ultrasound imaging of orthotopic tumors at day 12 post-surgery. Representative ultrasound images from orthotopic groups; the yellow line indicates pancreatic tissues and light blue indicates tumors.

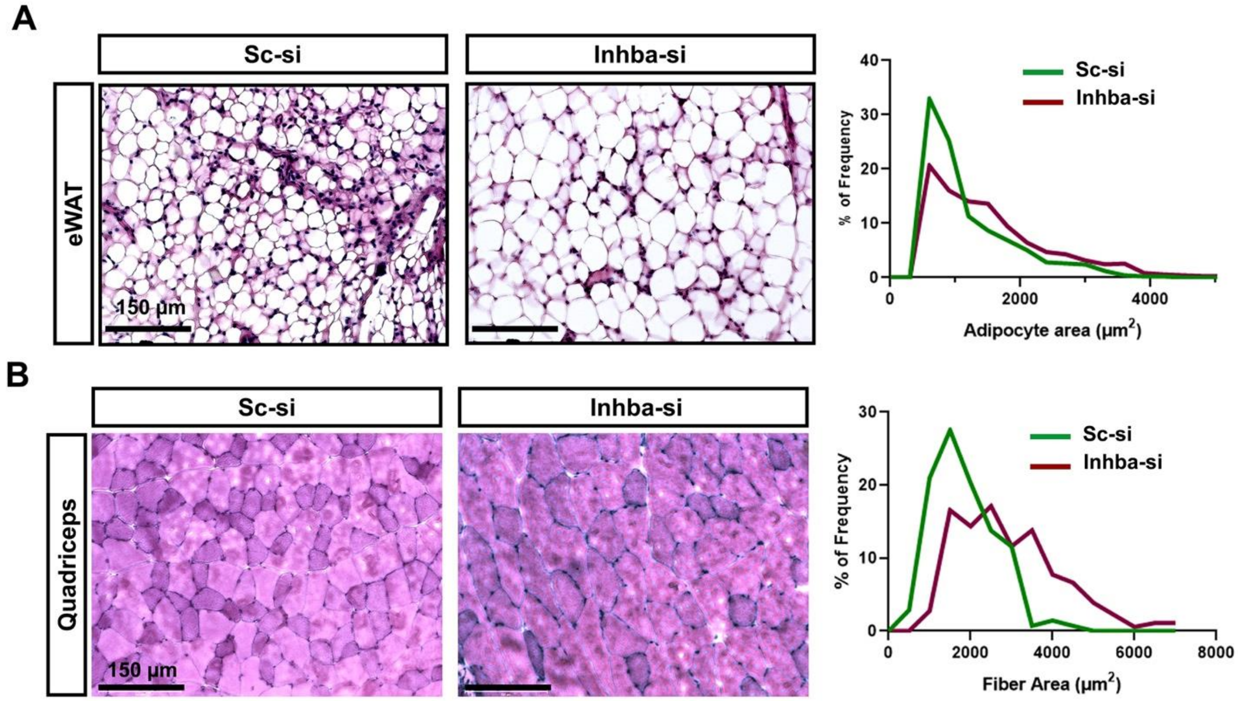

Figure S4. H&E staining of epididymal white adipose and quadriceps skeletal muscle tissue. A. Paraffin-embedded epididymal white adipose tissue section and size distribution of adipocytes. **B.** frozen section of quadriceps skeletal muscle tissue & size distribution of muscle fiber.

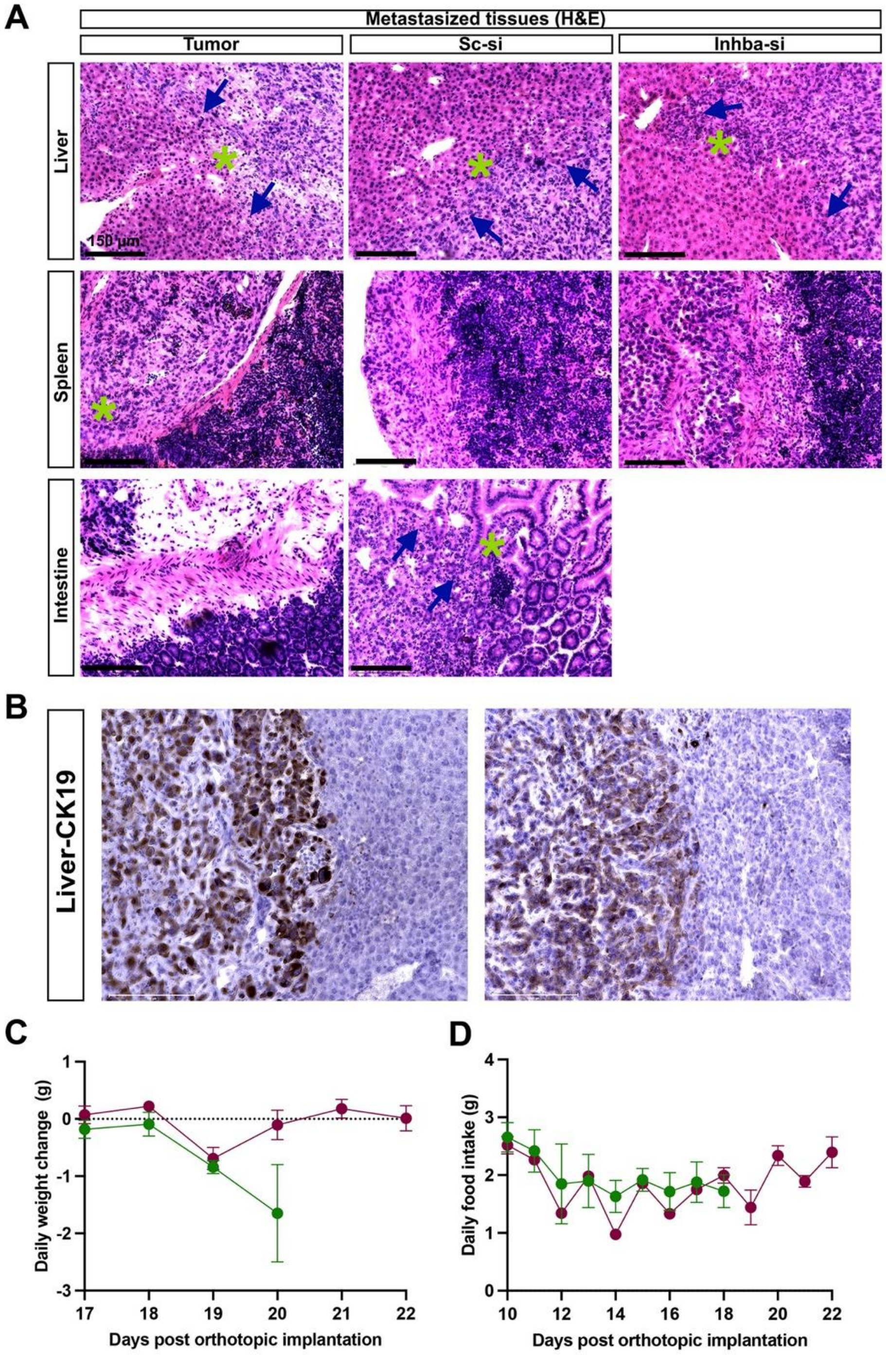

Figure S5. PDAC metastasis and changes in body weight and food intake during survival test. A. H&E staining of metastasized liver, spleen, and intestine to confirm invasive metastasis. Green asterisk indicates invasive metastasis. Blue arrow indicates invading cancer cells. **B.** Confirmation of invasive liver metastasis with IHC for CK19. **C and D.** Body weight change and food intake during survival test.

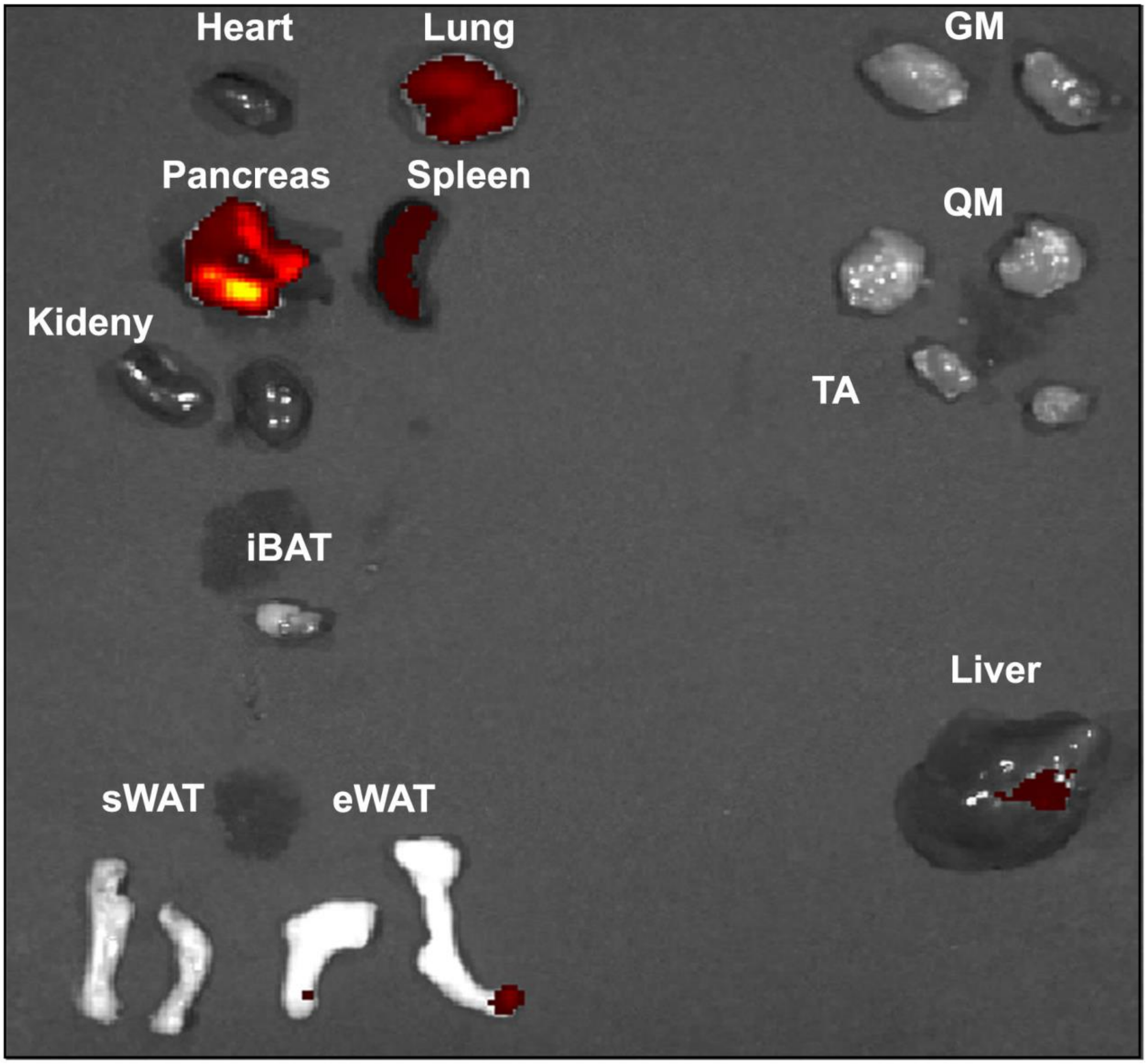

Figure S6. Biodistribution of Cy5.5-labeled siRNA-PCX nanoparticles *in vivo*. Representative whole image of accumulated Cy5.5 fluorescence in tissues taken by IVIS imaging; Interscapular brown adipose tissue (iBAT), subcutaneous white adipose tissue (sWAT), epididymal white adipose tissue (eWAT), gastrocnemius muscle (GM), quadriceps muscle (QM), and tibialis anterior muscle (TA).

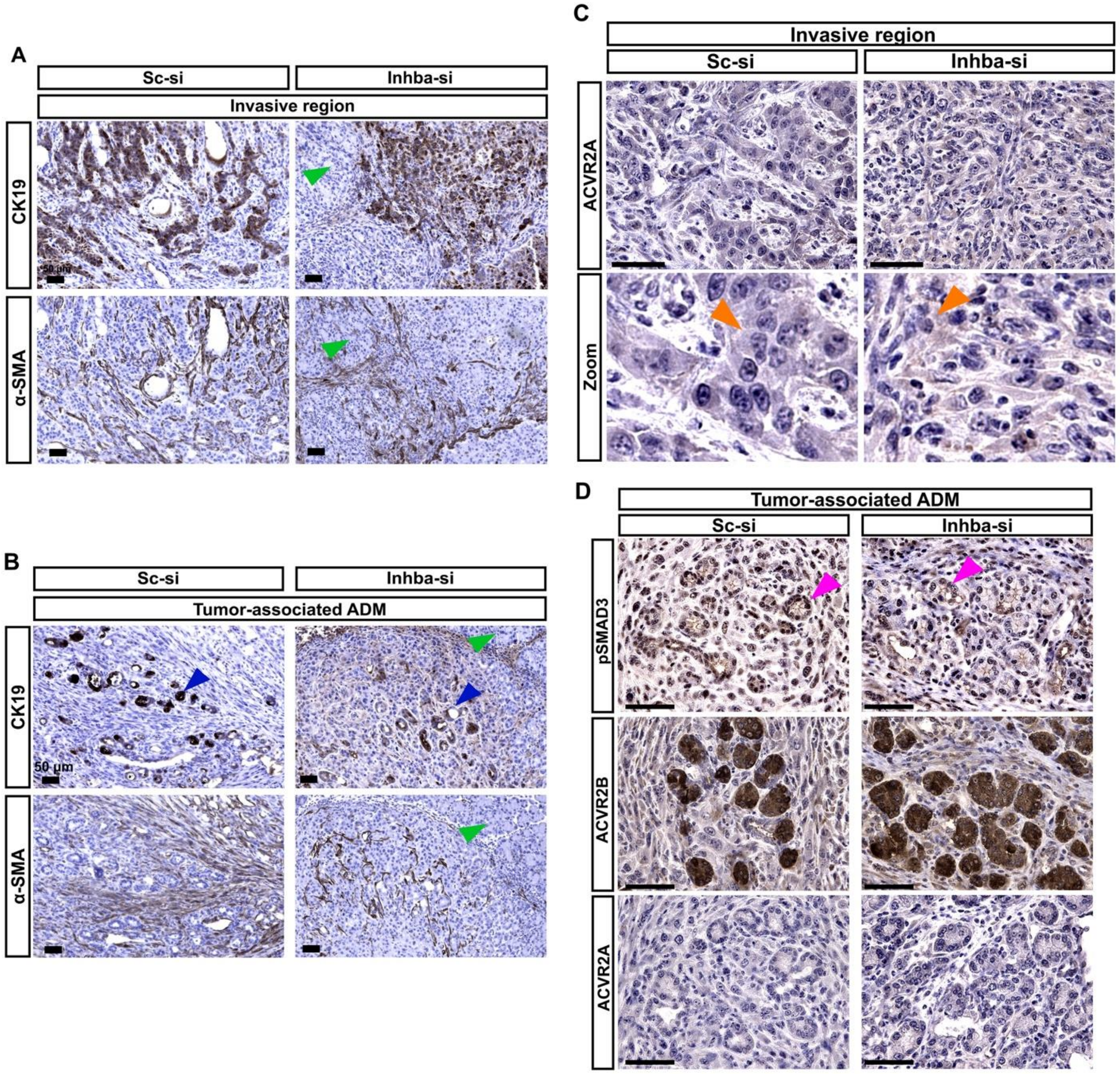

Figure S7. IHC of tumor CK19, α-SMA, ACVR2A, ACVR2B and pSMAD3 (Ser423/425). A and B. CK19 and α-SMA staining of invasive region and acinar-to-ductal transforming region of tumors. Green arrow indicates acinar cells. Blue arrow indicates duct-like cells. **C.** ACVR2A expression of invasive region of tumors by IHC. Orange arrow indicates invasive cancer cells. **D.** pSMAD3, ACVR2B and 2A staining of acinar-to-ductal transforming (pink arrow) region of tumors. Scale bar 50 μm.

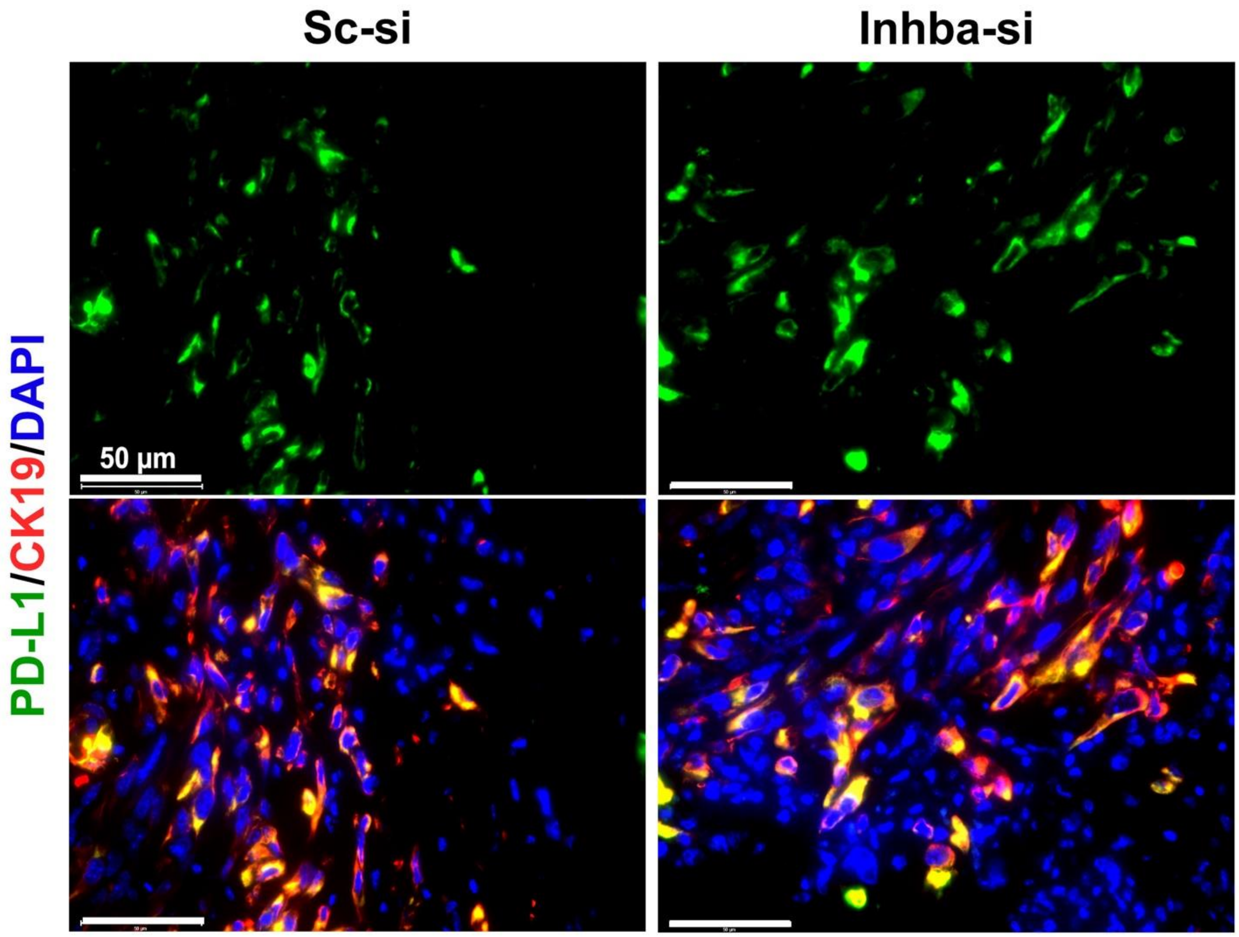

Figure S8. PD-L1 expression remained unchanged in CK19-positive cells in tumors by Inhba-si. Representative image of tumor immunofluorescence staining for PD-L1 and CK19 from mice treated with either Sc-si or Inhba-si. Scale bar = 50 μm.

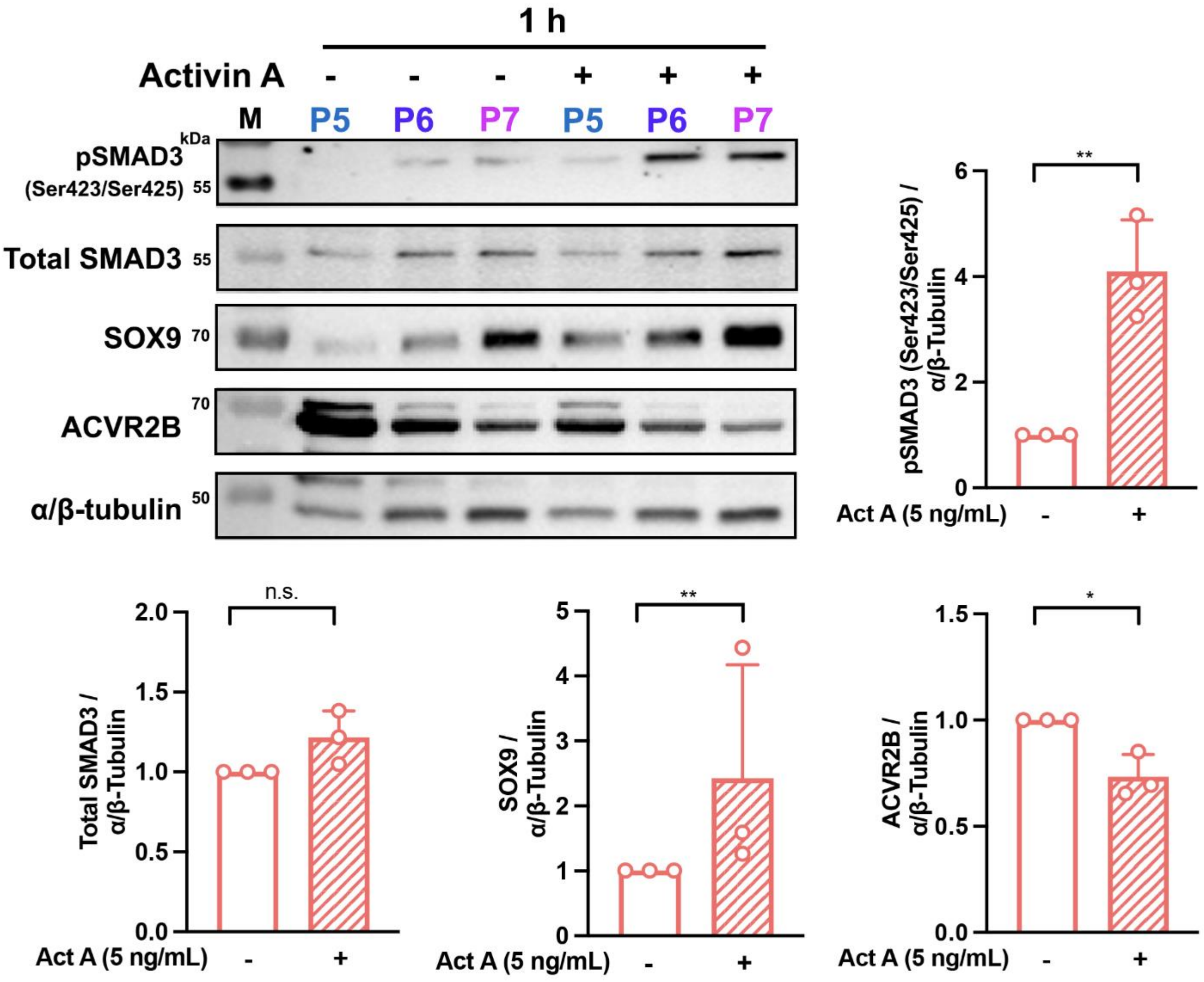

Figure S9. Activin A upregulates SOX9 and decreases ACVR2B in 266-6 acinar cells. Western blot analysis of pSMAD3, SMAD3, SOX9 and ACVR2B in 266-6 acinar cells treated with Activin A (5 ng/mL) for 1 hour (three independent experiments, n=3). * $P < 0.05$

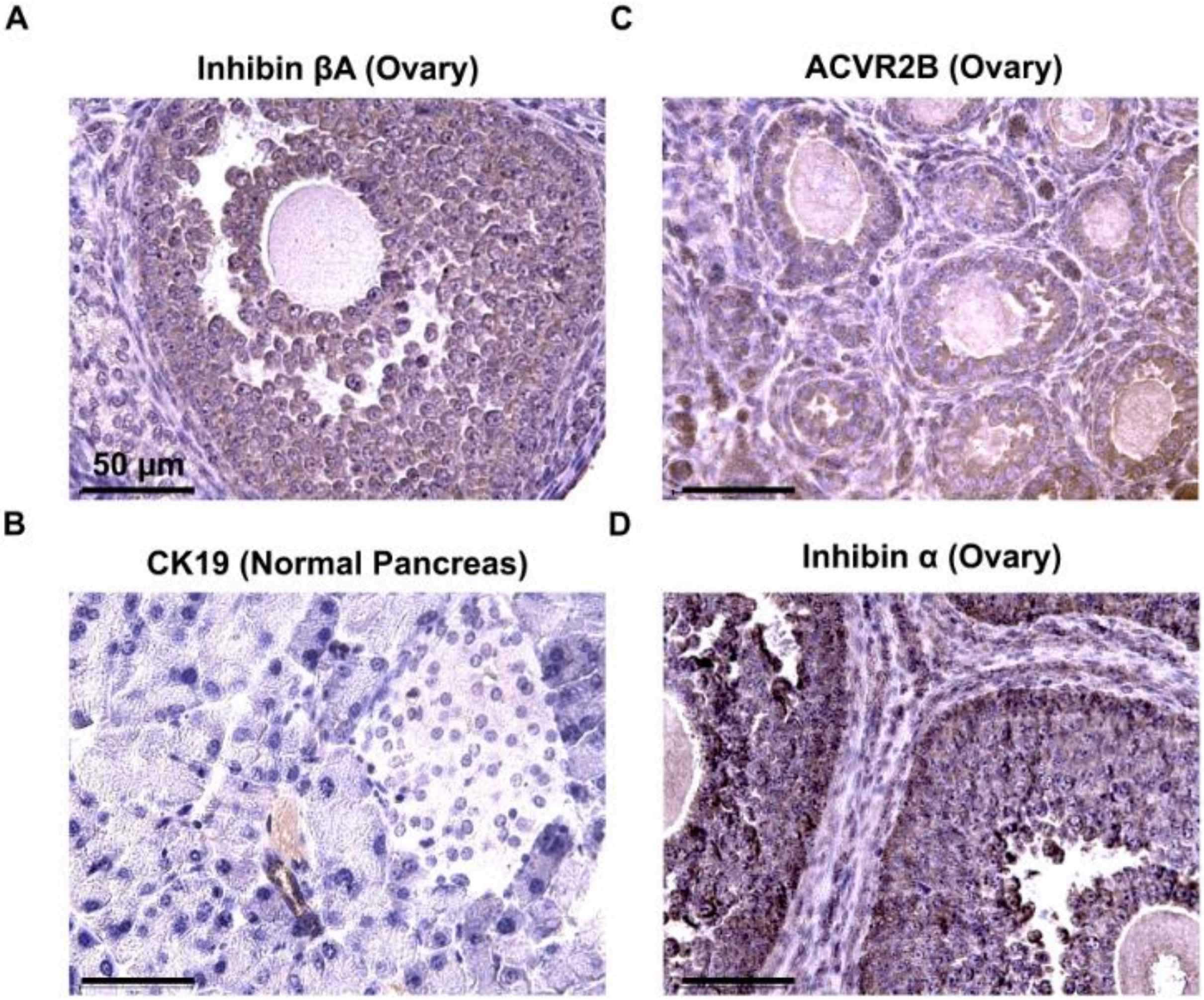

Figure S10. IHC assays of antibodies with positive control tissues. A. Intense staining of Inhibin βA on granulosa cells in antral follicles of mouse ovary. **B.** Duct-specific CK19 staining on the mouse pancreas. **C and D**. Intense staining of ACVR2B and Inhibin α on granulosa cells in growing and antral follicles of mouse ovary. Scale bar = 50 μm.

Legends for Supplementary Tables

Table S1. Chi-square test for tissue metastasis

Table S4. Chi-square test for tissue metastasis

Group	Metastasis (nodules) Yes	No	*p-value*
TO	8	8	*ns*
Sc-si	13	7	
TO	8	8	*<0.05*
Inhba-si	2	18	
Sc-si	13	7	*<0.001*
Inhba-si	2	18	

Table S2. Tissues with nodules and H&E-confirmed invasive metastasis

	Sham (n=4)	Tumor (n=4)	Sc-si (n=5)	Inhba-si (n=5)	Sham (n=4)	Tumor (n=4)	Sc-si (n=5)	Inhba-si (n=5)
	**Tissues with nodules**				**Invasive metastasis (H&E confirmed)**			
Liver	0	3 (75%)	5 (100%)	2 (40%)	0	3 (100%)	5 (100%)	1 (50%)
Spleen	0	2 (50%)	4 (80%)	0	0	0	0	0
Kidney	0	2 (50%)	3 (60%)	0	0	0	1 (33%)	0
Intestine	0	1 (25%)	1 (20%)	0	0	0	1 (100%)	0

Table S3. List of sequences for primers and siRNAs

Table S3. List of sequences for primers and siRNAs

Gene	Primer sequence	Species
Inhba	Mm00434339_m1	Mouse
Muc5b	s: 5’-GCATCATCAACAGTGCAACG-3’<br>as: 5’-GCAGAAACACTCGCAGTCA-3’	Mouse
Muc1	s: 5’-CCTACCATCCTATGAGTGAATACC-3’<br>as: 5’-GACTGCTACTGCCATTACCTG-3’	Mouse
Muc4	s: 5’-CAGCCTCTCCAAGAAATGTAGT-3’<br>as: 5’-GACAAGTTAGTCCTGACATCCC-3’	Mouse
Muc5ac	s: 5’-CCACTTTCTCCTTCTCCACACC-3’<br>as:5’-GGTTGTCG9ATGCAGCCTTGCTT-3’	Mouse
Acvr1a	s: 5’-CCATTGAAGGGCTCATCACCAC-3’<br>as: 5’-CCGTTCTTGTACCAGGAAAGG-3’	Mouse
Acvr1b	s: 5’-TTCTTCCCCCTTGTTGTCCTC-3’<br>as: 5’-ACAGGTGTAGTTGGTCTGTAGG-3’	Mouse
Acvr1c	s: 5’-AGACGGTGATGCTGAGACACGA-3’<br>as: 5’-GACCATTCCAGCCACAGTCACT-3’	Mouse
Acvr2a	s: 5’-TCATACCTGCATGAGGATGTG-3’<br>as: 5’-CCAAGCCAAAGTCAGCCA-3’	Mouse
Acvr2b	s: 5’-CCTGGCTGTTCGGTTTGAG-3’<br>as: 5’-GCGTCTCTCTGGAAGTTGATG-3’	Mouse
Rn18S	Mm03928990_g1	Mouse
#1 Inhba DsiRNA	5’-AACGCGAUCAGAAAGCUUCAUGUGG-3’<br>3’AGUUGCGCUAGUCUUUCGAAGUACACC-5’	Mouse
#2 Inhba DsiRNA	5’-CUGAAAGUCCCCAAGGCUAACAGAA-3’<br>3’-AGGACUUUCAGGGGUUCCGAUUGUCUU-5’	Mouse
#3 Inhba DsiRNA	5’-ACGAUGAUGGUCAAAACAUCAUCAA-3’<br>3’-AAUGCUACUACCAGUUUUGUAGUAGUU-5’	Mouse
#4 Inhba DsiRNA	5’-GUCAAGAAGCACAUCUUAAACAUGC-3’<br>3’-GACAGUUCUUCGUGUAGAAUUUGUACG-5’	Mouse
Scrambled negative control DsiRNA	Cat. # 51-01-19-09, Integrated DNA Technologies	Human, Mouse, Rat

Table S4. List of primary and secondary antibodies

Table S4. List of primary antibodies

Antibody	Host	Dilution	Company	Catalog no.
Inhibin βA	Rabbit	1:50	Gifted by Dr. Wylie Vale	
SOX9	Rabbit	1:1000	Cell Signaling Technology	82630S
Cytokeratin 19	Rat	1:100	Development Studies Hydridoma Bank	TROMA-III-c
Smad3	Rabbit	1:1000 or 1:50	Cell Signaling Technology	9523S
α-SMA	Rabbit	1:50	Cell Signaling Technology	19245T
Acvr2A	Rabbit	1:50	Millipore Sigma	HPA046997
Acvr2B	Rabbit	1:50	Invitrogen	PA5-111122
Inhibin α	Rabbit	1:50	Gifted by Dr. Wylie Vale	
Bax	Rabbit	1:50	Cell Signaling Technology	#2772
PD-L1	Rabbit	1:50	R&D Systems	AF1019
Prrx1	Rabbit	1:100	Thermo Fisher	PA5-106700
CD3	Rabbit	1:50	Cell Signaling Technology	78588T
CD8	Rabbit	1:50	Cell Signaling Technology	98941T
Amylase	Rabbit	1:50	Cell Signaling Technology	3796S
p-p38	Rabbit	1:1000	Cell Signaling Technology	9211S
p38	Rabbit	1:1000	Cell Signaling Technology	9212S
p-Smad3	Rabbit	1:1000	Rockland	600-401-919
E-Cadherin	Rabbit	1:50	Cell Signaling Technology	14472S
N-Cadherin	Rabbit	1:50	Cell Signaling Technology	13116S
CFTR	Rabbit	1:50	Novus Biologicals	NB300-511
α/β-Tubulin	Rabbit	1:1000	Cell Signaling Technology	2148S

List of secondary antibodies

Antibody	Conjugation	Dilution	Company	Catalog no.
Anti-rabbit IgG	HRP	1:2000	Cell Signaling Technology	7074
Anti-mouse IgG	HRP	1:2000	Cell Signaling Technology	7076
Anti-rabbit IgG	Biotinylated	1:200 or 1:400	Vector Laboratories	BA-1000-1.5
Anti-rat IgG	Biotinylated	1:200 or 1:400	Vector Laboratories	BA-9400-1.5
Anti-mouse IgG	Alexa Fluor 488	1:100-200	Invitrogen	A-11001
Anti-rabbit IgG	Alexa Fluor 488	1:100-200	Invitrogen	A-11008
Anti-rat IgG	Alexa Fluor 568	1:100-200	Invitrogen	A-11077
Anti-Goat IgG	Alexa Fluor 488	1:100	Invitrogen	A-11055
